# Characterization of orthopedic manifestations in patients with mucopolysaccharidosis II using data from 15 years of the Hunter Outcome Survey

**DOI:** 10.1002/jmd2.12401

**Published:** 2023-11-27

**Authors:** Bianca Link, Jaco Botha, Roberto Giugliani

**Affiliations:** ^1^ University Children's Hospital Zürich Switzerland; ^2^ Takeda Pharmaceuticals International AG Zürich Switzerland; ^3^ Department of Genetics/UFRGS Medical Genetics Service/HCPA, INAGEMP and Casa dos Raros Porto Alegre Brazil; ^4^ Dasa Genomica São Paulo Brazil

**Keywords:** Hunter Outcome Survey, Hunter syndrome, joint stiffness, mucopolysaccharidosis II, musculoskeletal, orthopedic

## Abstract

Mucopolysaccharidosis II (MPS II) is a rare, life‐limiting lysosomal storage disease caused by reduced iduronate‐2‐sulfatase activity. Patients experience broad ranging signs and symptoms, including bone and joint manifestations. This study reported on orthopedic involvement and management in patients with MPS II using 15 years of data from the Hunter Outcome Survey (HOS). Of the 245 patients in the study population, 90.2% had skeletal deformity (median onset, 2.8 years), 76.7% had upper body stiffness (onset, 4.2 years), and 61.2% had lower body stiffness (onset, 5.3 years); 63.7% of patients had at least three joint manifestations. Orthopedic manifestations were common in adults and children with MPS II, and in patients with and without cognitive impairment. Joint range of motion (JROM) was restricted in all joints assessed (shoulder, elbow, hip, wrist, knee, and ankle). Little correlation was observed between JROM measurements, subjective reports of joint stiffness and limited function, and 6‐minute walk test results. Patients with joint stiffness and limited function were generally more likely to have central and peripheral nervous system, pulmonary, and cardiovascular manifestations than those without these symptoms. Carpal tunnel decompression was the most common orthopedic surgery (recorded in 49/245 patients [20.0%]), but orthopedic surgeries were uncommon overall. Our findings highlight the need for routine monitoring of orthopedic manifestations using multiple assessment types in patients with MPS II to help inform clinical decision‐making and improve patient quality of life. They also underline the contribution of factors other than orthopedic manifestations to the walking ability of patients with MPS II.

**Synopsis**

Data from HOS indicate that orthopedic involvement is common in all patients with MPS II, and that multiple assessment types may be required to generate an accurate picture of the disease burden caused by orthopedic manifestations.

## 1 INTRODUCTION

Mucopolysaccharidosis II (MPS II; Hunter syndrome; OMIM 309900) is a rare, life‐limiting, X‐linked lysosomal storage disease. It is caused by reduced activity of the lysosomal enzyme iduronate‐2‐sulfatase (I2S), which is responsible for the degradation of some glycosaminoglycans (GAGs).^1^ Accumulation of GAGs throughout the body results in a multisystemic disease with a broad range of signs and symptoms, including coarse facial features, hepatosplenomegaly, respiratory and cardiac impairment, and joint stiffness leading to restricted movement.^2^, ^3^, ^4^ Around two thirds of patients also experience central nervous system (CNS) involvement, resulting in severe learning difficulties and neurological decline.^5^, ^6^

Treatment for MPS II is available in the form of enzyme replacement therapy (ERT) with intravenous recombinant I2S (idursulfase [marketed as Elaprase®]; Takeda Pharmaceuticals U.S.A. Inc., Lexington, MA, USA). Idursulfase has been shown to improve survival and to reduce the severity of many somatic disease manifestations, including reduced mobility, in patients with MPS II.^7^, ^8^, ^9^, ^10^, ^11^, ^12^, ^13^, ^14^, ^15^, ^16^ Intravenous idursulfase is not expected to treat cognitive decline owing to its inability to cross the blood–brain barrier.^7^, ^17^

The Hunter Outcome Survey (HOS) is a large, multicenter, observational registry funded by Takeda. Since 2005, it has collected long‐term data on the natural history of MPS II and the treatment of patients with intravenous idursulfase. A previous study described the orthopedic manifestations in patients with MPS II enrolled in HOS as of January 2009.^18^ Skeletal manifestations were common, with around 80% of patients affected, and a quarter of patients had abnormal gait. Furthermore, the presence of orthopedic manifestations was found to be associated with CNS and pulmonary manifestations.^18^ Individual tailored surgical and non‐surgical therapies are recommended for patients with orthopedic manifestations, given that ERT is unlikely to alleviate existing bone and joint manifestations fully.^18^, ^19^

The number of patients with orthopedic data available in HOS has almost doubled since the last publication on this topic in 2010.^18^ This study aims to report on additional findings around orthopedic involvement and management in MPS II as documented through 15 years of data collection in HOS, and to explore the relationship between joint function of the lower limb and walking ability in patients with MPS II.

## 2 MATERIALS AND METHODS

### 2.1 HOS registry design

Data are collected in HOS on patients with MPS II who are untreated or receiving treatment with intravenous idursulfase; patients who have received a bone marrow transplant can also be enrolled. Details of the HOS registry have been published previously.^2^, ^18^ Cognitive impairment is recorded in HOS based on the answer to the following question: “Cognitive impairment? Yes/No.” This may be informed by the clinical impression of the physician or by formal cognitive tests.

Independent Review Board/Ethics Committee approval was obtained for all participating centers before patient enrollment. Written informed consent was also obtained from each patient, their parents, or a legal representative.

### 2.2 Patient population

All male patients in HOS with data available on joint range of motion (JROM) as of July 23, 2021, were included in this analysis. Patients were only included if they had a signed informed consent form.

### 2.3 Descriptive statistics and data analyses

Data were summarized for all patients in the population who had the relevant information available in HOS (as of July 23, 2021).

For all assessments, the most recent one was used for untreated patients and the one closest to ERT initiation (no later than 91 days after ERT start) was used for treated patients to limit the confounding effects of their treatment. The presence or absence of joint stiffness and limited function and orthopedic manifestations was inferred from the latest entries in the HOS “signs and symptoms” and “medical history” forms before initiation of any ERT. For the analysis of upper and lower body stiffness, the “lower body” category consisted of the hip, knee, and ankle, and the “upper body” category consisted of the spine, shoulder, elbow, wrist, and hand.

JROM data were manually processed to exclude values that were considered impossible and to impute values for JROM described using free text or as a range. Normative numerical values were used when terms such as “free,” “full,” or “norm” were used on the case report forms for JROM. These values were obtained from the American Medical Association and the American Academy of Orthopedic Surgeons.^20^, ^21^ If a numerical range was provided but this had a lower value of zero, the upper limit of the range was used. Uninterpretable entries were recategorized as “missing.”

Six‐minute walk test (6MWT) results were reported for patients with normal and abnormal JROM assessments of the relevant lower body movements (knee extension and ankle dorsiflexion), and for patients with and without joint stiffness and limited function of the knee and ankle. 6MWT assessments were excluded if they were completed when the patient was younger than 5 years of age or if assistance was required to complete the test.

For the analysis of surgeries and procedures, the HOS medical monitor and authors identified categories in the database that were thought to be related to orthopedic manifestations. Orthopedic surgeries entered into the “other” category were reclassified into existing categories based on descriptions in the free‐text field. Surgeries and procedures considered to be orthopedic but not fitting into an existing orthopedic category were designated as “other.”

To test for correlations between joint stiffness and limited function and disease manifestations in other organ systems, Fisher's exact test was performed using data obtained before any ERT.

## 3 RESULTS

### 3.1 Patient characteristics

Patient characteristics for the study population (*N* = 245) are summarized in Table 1. Most of the patients were from Europe (183/245; 74.7% of patients) and 224/245 patients (91.4%) were under 18 years of age (hereafter referred to as “children”). The median (10th percentile [P10], 90th percentile [P90]) age at first symptom onset was 1.6 (0.1, 4.5) years and ERT was received by 228/245 patients (93.1%). The proportion of patients with cognitive impairment (101/245; 41.2%) was similar to those without cognitive impairment (144/245; 58.8%).

**TABLE 1:** Summary of patient characteristics.

	All patients (*N* = 245)
Region, *n* (%)	*n* = 245
Asia Pacific	4 (1.6)
Europe	183 (74.7)
Latin America	35 (14.3)
North America	23 (9.4)
Age at first symptom onset, years	*n* = 219
Mean (SD)	2.2 (2.0)
Median (P10, P90)	1.6 (0.1, 4.5)
Age at diagnosis, years	*n* = 243
Mean (SD)	5.0 (6.4)
Median (P10, P90)	3.9 (1.3, 8.0)
Age at JROM assessment,^a^ years	*n* = 245
Mean (SD)	11.9 (9.9)
Median (P10, P90)	8.9 (3.1, 25.9)
ERT treatment status, *n* (%)	*n* = 245
Treated	228 (93.1)
Untreated	17 (6.9)
Age at ERT start, years	*n* = 228
Mean (SD)	11.0 (9.3)
Median (P10, P90)	8.2 (2.8, 24.0)
Cognitive impairment before any ERT, *n* (%)	*n* = 245
Yes	101 (41.2)
No	144 (58.8)

Abbreviations: ERT, enzyme replacement therapy; JROM, joint range of motion; P10, 10th percentile; P90, 90th percentile; SD, standard deviation.

^a^ JROM measurements were taken from the assessment closest to ERT initiation that was no later than 91 days after ERT start for treated patients and the most recent assessment for untreated patients.

### 3.2 Orthopedic manifestations

Skeletal manifestations were documented in 221/245 patients (90.2%); the median (P10, P90) age at onset of first skeletal manifestation was 2.8 (0.4, 6.8) years. The five most common skeletal manifestations were coarse facial features (198/245; 80.8% of patients), claw hands (130/245; 53.1%), kyphosis/gibbus (88/245; 35.9%), scoliosis (45/245; 18.4%), and foot deformity (16/245; 6.5%).

Both upper and lower body stiffness were common and were present in 188/245 (76.7%) and 150/245 (61.2%) patients, respectively (Table 2). The median (P10, P90) age at earliest onset of stiffness was 4.2 (1.3, 13.7) years for the upper body and 5.3 (2.0, 17.8) years for the lower body.

**TABLE 2:** JROM measurements stratified by presence of joint stiffness and limited function in the relevant joints.

	Joint stiffness and limited function present		
Joint and movement	Yes	No	Normal JROM, °^,^ ^a^
Shoulder abduction, °	*n* = 136	*n* = 79	
Median JROM (P10, P90)	101.3 (70.0, 130.0)	102.5 (77.5, 150.0)	180
Elbow extension, °	*n* = 137	*n* = 85	
Median JROM (P10, P90)	−20.0 (−47.5, 25.0)	−9.0 (−36.0, 25.0)	0–10
Wrist extension, °	*n* = 85	*n* = 135
Median JROM (P10, P90)	32.5 (10.0, 63.5)	35.0 (8.0, 70.0)	60–70
Hip extension, °	*n* = 74	*n* = 122	
Median JROM (P10, P90)	0.0 (−22.5, 20.0)	6.8 (−17.5, 30.0)	20–30
Knee extension, °	*n* = 104	*n* = 104	
Median JROM (P10, P90)	−8.8 (−22.5, 10.0)	0.0 (−20.0, 22.5)	0–10
Ankle dorsiflexion, °	*n* = 73	*n* = 97	
Median JROM (P10, P90)	3.5 (−17.5, 20.0)	10.0 (−7.5, 22.5)	20

Abbreviations: AAOS, American Association of Orthopedic Surgeons; AMA, American Medical Association; JROM, joint range of motion; P10, 10th percentile; P90, 90th percentile.

^a^ Normal JROM defined according to a combination of the criteria developed by the AMA and the AAOS.^20^, ^21^

Most (192/245; 78.4%) patients had at least one joint manifestation, and there were high proportions of patients with 3–5 (73/245; 29.8%) or 6–8 (83/245; 33.9%) manifestations. The presence of stiffness and the number of joint manifestations were similar in all patients and children.

### 3.3 JROM

The median age at JROM measurement was 8.9 (3.1, 25.9) years, and JROM was generally lower than the normal range in the joint movements assessed (Table S1). An exception to this was shoulder extension, for which the median JROM was within the normal range. The most affected joint movements (median JROM >15° outside of the normal range) were hip extension, shoulder abduction, external rotation and flexion, elbow extension, and wrist extension. Almost all patients (229/245, 93.5%) had abnormal JROM in more than one joint.

There was a weak association between JROM measurements and joint stiffness and limited function assessments, with JROM found to be generally lower in patients with than in those without reported joint stiffness and limited function (Table S2). However, for shoulder abduction and wrist extension, JROM values for patients were similar with and without reported joint stiffness and limited function (101.3° vs. 102.5° for shoulder abduction and 32.5° vs. 35.0° for wrist extension, respectively). The median JROM measurements for patients both with and without reported joint stiffness and limited function were lower than the normal ranges for all movements except for knee extension.

### 3.4 Relationship of JROM and joint stiffness and limited function with 6MWT performance

The median (P10, P90) distance walked in the 6MWT was similar for patients with normal JROM for knee extension and ankle dorsiflexion compared with patients with abnormal JROM for these movements (390 m [280, 540 m], *n* = 29 vs. 381 m [208, 518 m], *n* = 49, respectively). Results were similar when patients with cognitive impairment (*n* = 90) were excluded (Table S3).

The median (P10, P90) distance walked in the 6MWT was similar for patients with and without joint stiffness and limited function of the knee and ankle (406 m [208, 540 m], *n* = 33 vs. 368 m [237, 510 m], *n* = 56). Results were similar when patients with cognitive impairment (*n* = 101) were excluded (Table S3).

### 3.5 Relationship between orthopedic manifestations and signs and symptoms in other organ systems

Patients with (subjectively assessed) joint stiffness and limited function were generally more likely to have cardiovascular, CNS, peripheral nervous system (PNS), and pulmonary manifestations than those without joint stiffness and limited function (Fisher's exact test: *p* ≤ 0.0002) (Table 3). The effect was particularly notable for cardiovascular and PNS manifestations. Potential associations between cardiovascular, CNS, PNS, and/or pulmonary manifestations and some other orthopedic manifestations were also observed (Table S4).

**TABLE 3:** Relationship between joint stiffness and limited function and other disease manifestations.

	Joint stiffness and limited function present	
	No	Yes
	*n* = 51	*n* = 194
Cardiovascular manifestations		
Yes, *n* (%)	18 (35.3)	162 (83.5)
No, *n* (%)	33 (64.7)	32 (16.5)
*p* value:^a^ < 0.0001		
CNS manifestations		
Yes, *n* (%)	16 (31.4)	118 (60.8)
No, *n* (%)	35 (68.6)	76 (39.2)
*p* value:^a^ 0.0002		
PNS manifestations		
Yes, *n* (%)	9 (17.6)	110 (56.7)
No, *n* (%)	42 (82.4)	84 (43.3)
*p* value:^a^ < 0.0001		
Pulmonary manifestations		
Yes, *n* (%)	23 (45.1)	143 (73.7)
No, *n* (%)	28 (54.9)	51 (26.3)
*p* value:^a^ 0.0002		

Abbreviations: CNS, central nervous system; PNS, peripheral nervous system.

^a^ *p* value calculated using Fisher's exact test.

### 3.6 Orthopedic surgeries and procedures

Most patients (213/245; 86.9%) in the study population had undergone at least one surgical procedure. Orthopedic surgeries were uncommon, recorded for 59/213 patients (27.7%) who underwent a total of 68 surgeries (Figure S1). Carpal tunnel decompression was the most common orthopedic surgery, recorded in 49/245 patients (20.0%).

## 4 DISCUSSION

Our study demonstrates that orthopedic involvement is high among patients with MPS II, and that many different joints are affected. Despite this, the proportion of patients who underwent orthopedic surgeries was low. Our data also suggest an association between the presence of joint stiffness and limited function and CNS, PNS, pulmonary system, and cardiovascular system manifestations. These data build on those from a previous HOS study of orthopedic manifestations in 124 patients, confirming and adding to the findings in a larger patient population (*N* = 245).^18^

Consistent with previous studies, nearly all patients with MPS II in this study experienced skeletal deformity and joint stiffness and limited function.^18^, ^22^ Both upper and lower body stiffness were present in more than 60% of the population, and the majority of patients had at least three joint manifestations. The prevalence of orthopedic manifestations was high among adults and children with MPS II, and in patients with and without cognitive impairment, highlighting the need for careful monitoring of orthopedic signs and symptoms throughout the life of all patients with MPS II.

Restricted range of motion was observed in almost all joints assessed, with the shoulder being the most affected joint. The most severely affected movement for all assessed joints except for the shoulder was extension, supporting previous findings from HOS.^18^ The wide range of joints and movements affected is likely to have a significant impact on mobility, and may have a negative impact on school and social life, self‐esteem, and mental health of patients with MPS II.^23^

We observed only a weak correlation between JROM measurements and subjective joint stiffness and limited function reports, and fewer patients had normal JROM (defined as abnormal JROM in one or no joints) than had no reported joint stiffness and limited function. This could be due to the inability of patients with MPS II to determine accurately whether their joints are stiff, owing to the progressive nature of their condition and their lack of experience of “normal” joint mobility. Patients with cognitive impairment may also find it more difficult to describe their experience, making it more difficult for the physician to make a judgment. This result was further supported by the finding that JROM measurements for patients without reported joint stiffness and limited function were generally lower than the normal range.

6MWT results were similar between patients with normal and abnormal JROM measurements for knee extension and ankle dorsiflexion, and between patients with and without subjective reports of joint stiffness and limited function, suggesting that other disease manifestations may affect walking ability more than lower joint function. For example, restrictions to arm movement may limit walking speed and stride,^24^ and cardiac and/or pulmonary manifestations affect the ability of patients to carry out sustained physical activity. These results further demonstrate the discordance between subjective reports of joint stiffness and limited function and other measures of joint functionality (JROM and 6MWT).

Our data also showed that orthopedic manifestations, including (subjectively assessed) joint stiffness and limited function, are associated with manifestations in other organ systems, including the CNS, PNS, pulmonary system, and cardiovascular system. This adds to previous findings that demonstrated a link between joint stiffness and limited function and CNS and pulmonary manifestations, but not cardiovascular manifestations,^18^ and reflects the severe disease burden in all organ systems in patients with CNS involvement. This difference in results may be explained by the larger patient population in this study.

Patients with MPS II often require surgical intervention,^25^ but orthopedic surgeries were recorded relatively infrequently in this population. However, surgery data were only reported for patients with JROM data and could be limited by data completeness in the HOS registry. Additionally, some severely affected patients may have met the criteria for surgery but not been operated on owing to their overall clinical condition and/or care needs. Carpal tunnel decompression was the most common orthopedic surgery reported, reinforcing previous recommendations that patients with MPS II should be regularly screened for carpal tunnel syndrome.^18^ Individualized monitoring of orthopedic symptoms is required to ensure that all patients with a clinical need are recommended for surgery. Physical therapies such as hydrotherapy, which can enhance strength and improve mobility, should also be considered to alleviate orthopedic issues in all patients independent of the possibility for surgical correction.^18^, ^19^, ^26^

The strengths of this study include the fact that it is the largest reported analysis of orthopedic manifestations, including JROM, in patients with MPS II, and the wide range of orthopedic and related data available within HOS. Limitations include those typical of registry‐based analyses, such as missing data for certain parameters and differences in the assessment methods used between sites. Additionally, some JROM measurements were not entered into HOS in the correct format and were either excluded or were used to impute a probable value, which may have influenced the JROM findings. Furthermore, it should be noted that the proportion of patients who had cognitive impairment (41.2%) was lower than has previously been reported for patients with MPS II.^5^, ^6^ This is likely to be due to the exclusion of patients who did not have JROM measurements from this analysis, but it means that our findings may be skewed toward the non‐neuronopathic population. Assessments of treated patients were limited to those that took place closest to ERT initiation (no later than 91 days after ERT start); this means that the long‐term care needs of treated patients could not be assessed. There were also limited data available on changes in disease manifestations following treatment with idursulfase, precluding an analysis of treatment effects.

Our findings highlight the prevalence, range, and severity of orthopedic manifestations in patients with MPS II, and the need for routine monitoring of these manifestations in all patients to identify individuals who would benefit from surgery and/or physical therapy. Our observation that subjective reports of joint stiffness and limited function do not always reflect other measures of joint functionality emphasizes the importance of conducting multiple types of assessment for complete evaluation of orthopedic MPS II manifestations, so that patients can receive appropriate care and support.

## AUTHOR CONTRIBUTIONS

Bianca Link, Jaco Botha, and Roberto Giugliani all made substantial contributions to the conception of the work and the analysis and interpretation of data. All authors contributed to the drafting and revision of the manuscript and have read and approved the final version for submission. All authors agree to be accountable for their contributions.

## FUNDING INFORMATION

This study was sponsored and funded by Takeda Pharmaceutical International AG. Collection and analysis of data in HOS is supported by Takeda. Data analyses were performed by Takeda and under the direction of the authors. Medical writing support was provided under the direction of the authors and funded by Takeda Development Center Americas, Inc.

## CONFLICT OF INTEREST STATEMENT

Bianca Link has received consulting fees, fees for service, speaker fees, and/or travel expenses from BioMarin Pharmaceutical, Chiesi Farmaceutici, Sanofi Genzyme, and Takeda. Jaco Botha is a full‐time employee of Takeda and stockholder of Takeda Pharmaceuticals Company Limited. Roberto Giugliani has received consulting fees, fees for service, speaker fees, and/or travel expenses from and/or participated in advisory boards for Abeona Therapeutics, Alnylam Pharmaceuticals, Amicus Therapeutics, BioMarin Pharmaceutical, Chiesi Farmaceutici, Inventiva Pharma, Janssen Pharmaceuticals, JCR Pharmaceuticals, Novartis, Orphan Disease Center, Praxis Precision Medicines, PTC Therapeutics, REGENXBIO, Sanofi Genzyme, Sigilon Therapeutics, Swedish Orphan Biovitrum, Takeda, and Ultragenyx. He has performed contracted research for or received research grants from Allievex, Amicus Therapeutics, BioMarin Pharmaceutical, GC Pharma, Idorsia, Janssen Pharmaceuticals, JCR Pharmaceuticals, Lysogene, Sanofi Genzyme, Takeda, and Ultragenyx.

## ETHICS STATEMENT

Independent Review Board/Ethics Committee approval was obtained for all participating centers. HOS is conducted in accordance with Good Pharmacoepidemiological Practices (GPP), Good Research for Comparative Effectiveness principles, and the relevant principles of the International Conference on Harmonisation (ICH) Good Clinical Practice (GCP) guidelines (ICH E6).

## PATIENT CONSENT STATEMENT

Each patient, their parents, or a legal representative provided signed and dated written informed consent for participation in HOS. All patient information is managed in accordance with national data protection standards.

## Supporting information


**DATA S1:** Supporting information.

## Data Availability

The datasets, including the redacted study protocol, redacted statistical analysis plan, and individual participants data supporting the results reported in this article, will be made available within 3 months from initial request, to researchers who provide a methodologically sound proposal. The data will be provided after its de‐identification, in compliance with applicable privacy laws, data protection and requirements for consent and anonymization.

## References

[1] Muenzer J . Overview of the mucopolysaccharidoses. Rheumatology. 2011;50:v4‐v12. 22210669 10.1093/rheumatology/ker394

[2] Wraith JE , Beck M , Giugliani R , Clarke J , Martin R , Muenzer J . Initial report from the Hunter Outcome Survey. Genet Med. 2008;10:508‐516. 18580692 10.1097/gim.0b013e31817701e6

[3] Khan SA , Peracha H , Ballhausen D , et al. Epidemiology of mucopolysaccharidoses. Mol Genet Metab. 2017;121:227‐240. 28595941 10.1016/j.ymgme.2017.05.016 PMC5653283

[4] D'Avanzo F , Rigon L , Zanetti A , et al. Mucopolysaccharidosis type II: one hundred years of research, diagnosis, and treatment. Int J Mol Sci. 2020;21:1258. 32070051 10.3390/ijms21041258 PMC7072947

[5] Young ID , Harper PS , Newcombe RG , Archer IM . A clinical and genetic study of Hunter's syndrome. 2. Differences between the mild and severe forms. J Med Genet. 1982;19:408‐411. 6818348 10.1136/jmg.19.6.408 PMC1048951

[6] Burton BK , Giugliani R . Diagnosing Hunter syndrome in pediatric practice: practical considerations and common pitfalls. Eur J Pediatr. 2012;171:631‐639. 22383073 10.1007/s00431-012-1703-y PMC3306562

[7] Muenzer J , Wraith JE , Beck M , et al. A phase II/III clinical study of enzyme replacement therapy with idursulfase in mucopolysaccharidosis II (Hunter syndrome). Genet Med. 2006;8:465‐473. 16912578 10.1097/01.gim.0000232477.37660.fb

[8] Muenzer J , Beck M , Eng CM , et al. Long‐term, open‐labeled extension study of idursulfase in the treatment of Hunter syndrome. Genet Med. 2011;13:95‐101. 21150784 10.1097/GIM.0b013e3181fea459

[9] Broomfield A , Davison J , Roberts J , et al. Ten years of enzyme replacement therapy in paediatric onset mucopolysaccharidosis II in England. Mol Genet Metab. 2020;129:98‐105. 31383595 10.1016/j.ymgme.2019.07.016

[10] Burton BK , Jego V , Mikl J , Jones SA . Survival in idursulfase‐treated and untreated patients with mucopolysaccharidosis type II: data from the Hunter Outcome Survey (HOS). J Inherit Metab Dis. 2017;40:867‐874. 28887757 10.1007/s10545-017-0075-x

[11] Dalmau Serra J , Vitoria Minana I , Calderon Fernandez R , et al. Clinical response to long term enzyme replacement treatment in children, adolescent and adult patients with Hunter syndrome. Med Clin (Barc). 2015;145:392‐398. 26360015 10.1016/j.medcli.2015.06.015

[12] Lampe C , Bosserhoff A‐K , Burton BK , et al. Long‐term experience with enzyme replacement therapy (ERT) in MPS II patients with a severe phenotype: an international case series. J Inherit Metab Dis. 2014;37:823‐829. 24596019 10.1007/s10545-014-9686-7 PMC4158409

[13] Parini R , Rigoldi M , Tedesco L , et al. Enzymatic replacement therapy for Hunter disease: up to 9 years experience with 17 patients. Mol Genet Metab Rep. 2015;3:65‐74. 26937399 10.1016/j.ymgmr.2015.03.011 PMC4750582

[14] Tomanin R , Zanetti A , D'Avanzo F , et al. Clinical efficacy of enzyme replacement therapy in paediatric Hunter patients, an independent study of 3.5 years. Orphanet J Rare Dis. 2014;9:129. 25231261 10.1186/s13023-014-0129-1 PMC4180060

[15] Muenzer J , Giugliani R , Scarpa M , Tylki‐Szymańska A , Jego V , Beck M . Clinical outcomes in idursulfase‐treated patients with mucopolysaccharidosis type II: 3‐year data from the Hunter Outcome Survey (HOS). Orphanet J Rare Dis. 2017;12:161. 28974237 10.1186/s13023-017-0712-3 PMC5627440

[16] Muenzer J , Botha J , Harmatz P , Giugliani R , Kampmann C , Burton BK . Evaluation of the long‐term treatment effects of intravenous idursulfase in patients with mucopolysaccharidosis II (MPS II) using statistical modeling: data from the Hunter Outcome Survey (HOS). Orphanet J Rare Dis. 2021;16:456. 34717704 10.1186/s13023-021-02052-4 PMC8557006

[17] Whiteman DA , Kimura A . Development of idursulfase therapy for mucopolysaccharidosis type II (Hunter syndrome): the past, the present and the future. Drug Des Devel Ther. 2017;11:2467‐2480. 10.2147/DDDT.S139601 PMC5574592 28860717

[18] Link B , de Camargo Pinto LL , Giugliani R , et al. Orthopedic manifestations in patients with mucopolysaccharidosis type II (Hunter syndrome) enrolled in the Hunter Outcome Survey. Orthop Rev (Pavia). 2010;2:e16. 21808707 10.4081/or.2010.e16 PMC3143973

[19] Scarpa M , Almassy Z , Beck M , et al. Mucopolysaccharidosis type II: European recommendations for the diagnosis and multidisciplinary management of a rare disease. Orphanet J Rare Dis. 2011;6:72. 22059643 10.1186/1750-1172-6-72 PMC3223498

[20] AMA , ed. American Medical Association Guides to Evaluation of Permanent Impairment. 3rd ed, Revised ed. AMA Press; 1990.

[21] AAOS . Joint Motion: Method of Measuring and Recording. American Academy of Orthopedic Surgeons; 1965.

[22] Alkhzouz C , Cabau G , Lazea C , et al. Skeletal abnormalities and VDR1 gene polymorphisms in mucopolysaccharidosis patients. Pharmgenomics Pers Med. 2021;14:349‐358. 33889011 10.2147/PGPM.S295241 PMC8056862

[23] Hendriksz CJ , Berger KI , Lampe C , et al. Health‐related quality of life in mucopolysaccharidosis: looking beyond biomedical issues. Orphanet J Rare Dis. 2016;11:119. 27561270 10.1186/s13023-016-0503-2 PMC5000418

[24] Takami A , Cavan S , Makino M . Effects of arm swing on walking abilities in healthy adults restricted in the Wernicke‐Mann's limb position. J Phys Ther Sci. 2020;32:502‐505. 32884170 10.1589/jpts.32.502 PMC7443538

[25] Mendelsohn NJ , Harmatz P , Bodamer O , et al. Importance of surgical history in diagnosing mucopolysaccharidosis type II (Hunter syndrome): data from the Hunter Outcome Survey. Genet Med. 2010;12:816‐822. 21045710 10.1097/GIM.0b013e3181f6e74d

[26] Nan H , Park C , Maeng S . Mucopolysaccharidoses I and II: brief review of therapeutic options and supportive/palliative therapies. Biomed Res Int. 2020;2020:1‐18. 10.1155/2020/2408402 PMC7732385 33344633

