# Supplementary material for: Characterization of orthopedic manifestations in patients with mucopolysaccharidosis II using data from 15 years of the Hunter Outcome Survey
Source: JIMD Rep. 2023 Nov 27;65(1):17–24. doi: 10.1002/jmd2.12401 (PMC10764199; doi:10.1002/jmd2.12401)
Supplement: Supplementary file 1 — DATA S1: Supporting information. [file JMD2-65-17-s001.docx]

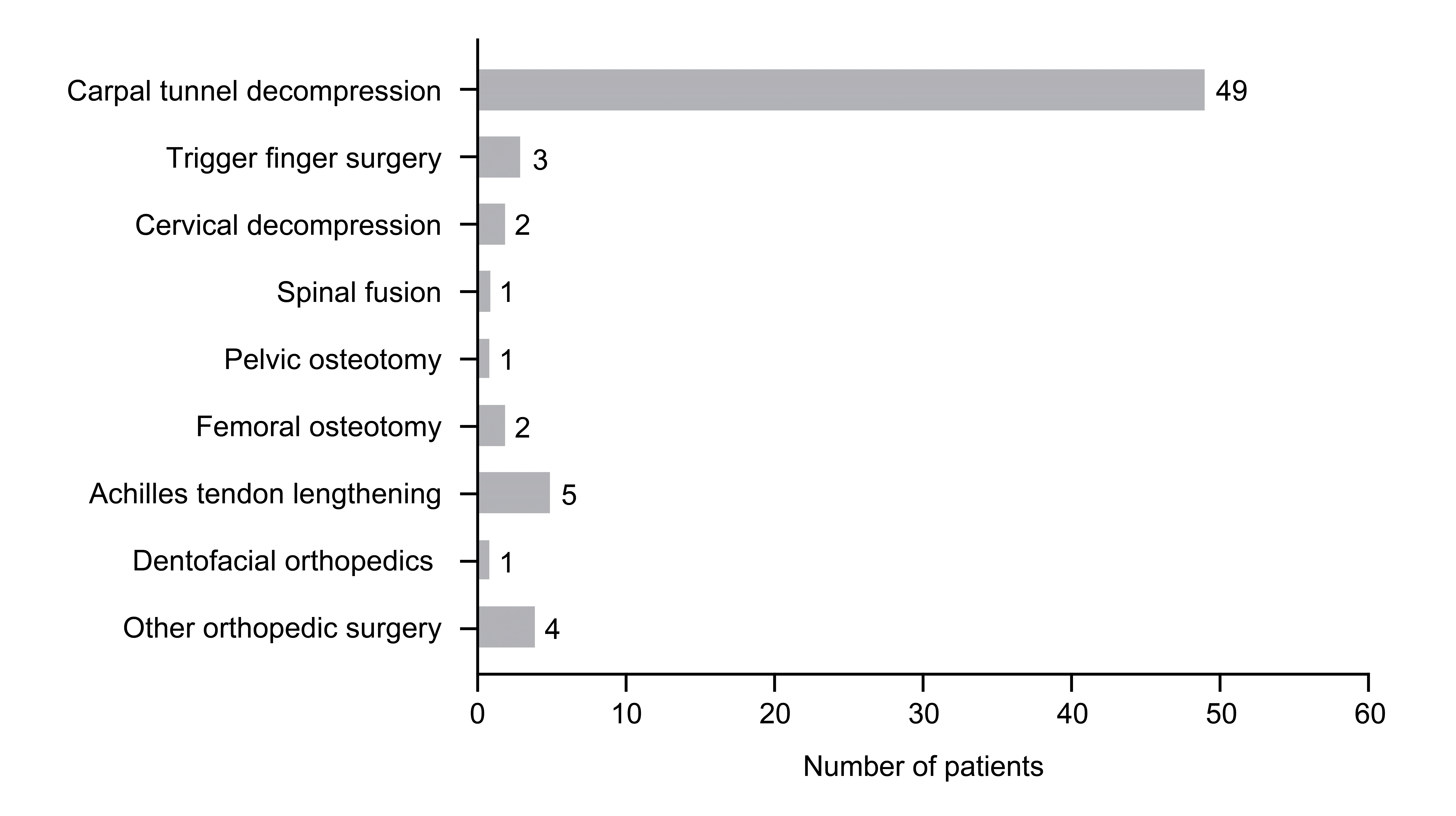


**FIGURE S1** Prevalence of orthopedic surgeries and procedures (*N* = 245).

*Note:* Surgeries are only included if they were carried out before ERT. Those with completely unknown dates are assumed to have occurred on or before baseline and are included in the graph.

ERT, enzyme replacement therapy.

**TABLE S1** JROM measurements.

| **Joint and movement** | **n^a^** | **Median (P10, P90) JROM, °** | **Normal JROM, °^,b^** | **Median JROM below normal JROM? Yes/No** |
| --- | --- | --- | --- | --- |
| Shoulder abduction | 215 | 102.5  (75.5, 140.0) | 180 | Yes |
| Shoulder extension | 134 | 54.3  (32.5, 80.0) | 50–60 | No |
| Shoulder external rotation | 168 | 60.0  (30.0, 89.0) | 90 | Yes |
| Shoulder flexion | 215 | 120.0  (90.0, 155.0) | 150–180 | Yes |
| Shoulder internal rotation | 160 | 61.3  (36.8, 88.0) | 70–90 | Yes |
| Elbow extension | 222 | −15.8  (−45.0, 25.0) | 0–10 | Yes |
| Wrist extension | 220 | 34.8  (9.0, 70.0) | 60–70 | Yes |
| Hip abduction | 171 | 34.5 (22.5, 52.5) | 40 | Yes |
| Hip extension | 196 | 3.0 (−20.0, 30.0) | 20–30 | Yes |
| Knee extension | 208 | −2.5  (−21.0, 12.5) | 0–10 | Yes |
| Ankle dorsiflexion | 170 | 5.3  (−10.0, 21.8) | 20 | Yes |

Abbreviation: AMA, American Medical Association; AAOS, American Association of Orthopedic Surgeons; JROM, joint range of motion; P10, 10th percentile; P90, 90th percentile.

^a^Number of patients for whom data were available for that variable. ^b^Normal JROM defined according to a combination of the criteria developed by the AMA and the AAOS.^20,21^

**TABLE S2** JROM measurements stratified by presence of joint stiffness and limited function in the relevant joints.

|  | **Joint stiffness and limited function present** | | **Normal JROM, °^,a^** |
| --- | --- | --- | --- |
| **Joint and movement** | **Yes** | **No** |  |
| Shoulder abduction, ° | *n* = 136 | *n* = 79 | 180 |
| Median JROM  (P10, P90) | 101.3  (70.0, 130.0) | 102.5  (77.5, 150.0) |  |
| Elbow extension, ° | *n* = 137 | *n* = 85 | 0–10 |
| Median JROM  (P10, P90) | −20.0  (−47.5, 25.0) | −9.0  (−36.0, 25.0) |  |
| Wrist extension, ° | *n* = 85 | *n* = 135 | 60–70 |
| Median JROM  (P10, P90) | 32.5 (10.0, 63.5) | 35.0  (8.0, 70.0) |  |
| Hip extension, ° | *n* = 74 | *n* = 122 | 20–30 |
| Median JROM  (P10, P90) | 0.0  (−22.5, 20.0) | 6.8  (−17.5, 30.0) |  |
| Knee extension, ° | *n* = 104 | *n* = 104 | 0–10 |
| Median JROM  (P10, P90) | −8.8  (−22.5, 10.0) | 0.0  (−20.0, 22.5) |  |
| Ankle dorsiflexion, ° | *n* = 73 | *n* = 97 | 20 |
| Median JROM  (P10, P90) | 3.5  (−17.5, 20.0) | 10.0  (−7.5, 22.5) |  |

Abbreviations: AMA, American Medical Association; AAOS, American Association of Orthopedic Surgeons; JROM, joint range of motion; P10, 10th percentile; P90, 90th percentile.

^a^Normal JROM defined according to a combination of the criteria developed by the AMA and the AAOS.^20,21^

**TABLE S3** 6MWT results stratified by JROM result or joint stiffness and limited function and cognitive status.

|  | **JROM assessment** | | **Joint stiffness and limited function assessment of the lower body^a^** | |
| --- | --- | --- | --- | --- |
| **Cognitive impairment at any time** | **Normal**  *n* = 24 | **Abnormal**  *n* = 66 | **No**  *n* = 50 | **Yes**  *n* = 51 |
| Yes, median (P10, P90) | 407  (219, 527) | 331  (192, 467) | 283  (206, 504) | 399  (178, 450) |
| No, median  (P10, P90) | 390  (288, 540) | 390  (268, 540) | 380  (285, 510) | 436  (280, 540) |

Abbreviations: 6MWT, 6-minute walk test; JROM, joint range of movement; P10, 10th percentile; P90, 90th percentile.

^a^Movements assessed were knee extension and ankle dorsiflexion.

**TABLE S4** Relationship between orthopedic manifestations and other disease manifestations.

|  | **Any skeletal deformity** | | **Claw hands** | | **Coarse facial features^a^** | | **Foot deformity** | | **Kyphosis/gibbus** | | **Scoliosis** | | **Shortening of the Achilles tendon** | | **Trigger finger** | |
| --- | --- | --- | --- | --- | --- | --- | --- | --- | --- | --- | --- | --- | --- | --- | --- | --- |
|  | **No** | **Yes** | **No** | **Yes** | **No** | **Yes** | **No** | **Yes** | **No** | **Yes** | **No** | **Yes** | **No** | **Yes** | **No** | **Yes** |
| *n* | 24 | 221 | 115 | 130 | 47 | 198 | 229 | 16 | 157 | 88 | 200 | 45 | 233 | 12 | 239 | 6 |
| Cardiovascular manifestations |  |  |  |  |  |  |  |  |  |  |  |  |  |  |  |  |
| Yes, *n* (%) | 6 (25.0) | 174 (78.7) | 66 (57.4) | 114 (87.7) | 22 (46.8) | 158 (79.8) | 164 (71.6) | 16 (100.0) | 103 (65.6) | 77 (87.5) | 138 (69.0) | 42 (93.3) | 171 (73.4) | 9 (75.0) | 174 (72.8) | 6 (100.0) |
| No, *n* (%) | 18 (75.0) | 47 (21.3) | 49 (42.6) | 16 (12.3) | 25 (53.2) | 40 (20.2) | 65 (28.4) | 0  (0) | 54 (34.4) | 11 (12.5) | 62 (31.0) | 3 (6.7) | 62 (26.6) | 3 (25.0) | 65 (27.2) | 0  (0) |
| *P* value^b^ | < .0001 | | < .0001 | | < .0001 | | .0079 | | .0002 | | .0006 | | 1.0000 | | .3458 | |
| CNS manifestations |  |  |  |  |  |  |  |  |  |  |  |  |  |  |  |  |
| Yes, *n* (%) | 5 (20.8) | 129 (58.4) | 51 (44.3) | 83 (63.8) | 12 (25.5) | 122 (61.6) | 124 (54.1) | 10 (62.5) | 73 (46.5) | 61 (69.3) | 107 (53.5) | 27 (60.0) | 125 (53.6) | 9 (75.0) | 130 (54.4) | 4 (66.7) |
| No, *n* (%) | 19 (79.2) | 92 (41.6) | 64 (55.7) | 47 (36.2) | 35 (74.5) | 76 (38.4) | 105 (45.9) | 6 (37.5) | 84 (53.5) | 27 (30.7) | 93 (46.5) | 18 (40.0) | 108 (46.4) | 3 (25.0) | 109 (45.6) | 2 (33.3) |
| *P* value^b^ | .0008 | | .0030 | | < .0001 | | .6087 | | .0008 | | .5081 | | .2339 | | .6920 | |
| PNS manifestations |  |  |  |  |  |  |  |  |  |  |  |  |  |  |  |  |
| Yes, *n* (%) | 3 (12.5) | 116 (52.5) | 37 (32.2) | 82 (63.1) | 13 (27.7) | 106 (53.5) | 108 (47.2) | 11 (68.8) | 64 (40.8) | 55 (62.5) | 87 (43.5) | 32 (71.1) | 110 (47.2) | 9 (75.0) | 113 (47.3) | 6 (100.0) |
| No, *n* (%) | 21 (87.5) | 105 (47.5) | 78 (67.8) | 48 (36.9) | 34 (72.3) | 92 (46.5) | 121 (52.8) | 5 (31.3) | 93 (59.2) | 33 (37.5) | 113 (56.5) | 13 (28.9) | 123 (52.8) | 3 (25.0) | 126 (52.7) | 0  (0) |
| *P* value^b^ | .0002 | | < .0001 | | .0019 | | .1219 | | .0014 | | .0009 | | .0773 | | .0123 | |
| Pulmonary manifestations |  |  |  |  |  |  |  |  |  |  |  |  |  |  |  |  |
| Yes, *n* (%) | 5 (20.8) | 161 (72.9) | 61 (53.0) | 105 (80.8) | 17 (36.2) | 149 (75.3) | 155 (67.7) | 11 (68.8) | 100 (63.7) | 66 (75.0) | 128 (64.0) | 38 (84.4) | 158 (67.8) | 8 (66.7) | 161 (67.4) | 5 (83.3) |
| No, *n* (%) | 19 (79.2) | 60 (27.1) | 54 (47.0) | 25 (19.2) | 30 (63.8) | 49 (24.7) | 74 (32.3) | 5 (31.3) | 57 (36.3) | 22 (25.0) | 72 (36.0) | 7 (15.6) | 75 (32.2) | 4 (33.3) | 78 (32.6) | 1 (16.7) |
| *P* value^b^ | < 0.0001 | | < 0.0001 | | < 0.0001 | | 1.0000 | | 0.0872 | | 0.0079 | | 1.0000 | | 0.6672 | |

Abbreviations: CNS, central nervous system; PNS, peripheral nervous system.

^a^Coarse facial features were classified as orthopedic manifestations because they are thought to be caused by a combination of facial bone dysostosis and the accumulation of glycosaminoglycans in the surrounding soft tissue.

^b^*P* value calculated using Fisher’s exact test.
